# On-site processing of single chromosomal DNA molecules using optically driven microtools on a microfluidic workbench

**DOI:** 10.1038/s41598-021-87238-3

**Published:** 2021-04-12

**Authors:** Akihito Masuda, Hidekuni Takao, Fusao Shimokawa, Kyohei Terao

**Affiliations:** 1 Department of Intelligent Mechanical Systems Engineering, Kagawa University, Takamatsu, 761-0396 Japan; 2 Nano-Micro Structure Device Integrated Research Center, Kagawa University, Takamatsu, 761-0396 Japan

**Keywords:** Biotechnology, Biomedical engineering

## Abstract

We developed optically driven microtools for processing single biomolecules using a microfluidic workbench composed of a microfluidic platform that functions under an optical microscope. The optically driven microtools have enzymes immobilized on their surfaces, which catalyze chemical reactions for molecular processing in a confined space. Optical manipulation of the microtools enables them to be integrated with a microfluidic device for controlling the position, orientation, shape of the target sample. Here, we describe the immobilization of enzymes on the surface of microtools, the microfluidics workbench, including its microtool storage and sample positioning functions, and the use of this system for on-site cutting of single chromosomal DNA molecules. We fabricated microtools by UV lithography with SU-8 and selected ozone treatments for immobilizing enzymes. The microfluidic workbench has tool-stock chambers for tool storage and micropillars to trap and extend single chromosomal DNA molecules. The DNA cutting enzymes DNaseI and DNaseII were immobilized on microtools that were manipulated using optical tweezers. The DNaseI tool shows reliable cutting for on-site processing. This pinpoint processing provides an approach for analyzing chromosomal DNA at the single-molecule level. The flexibility of the microtool design allows for processing of various samples, including biomolecules and single cells.

## Introduction

Biomolecules, including nucleic acids and proteins, are generally processed in bulk in solution by using enzymes to catalyze chemical reactions in standard biological assays. These processes are based on the stochastic binding of molecules driven by thermal diffusion and are nonspecific molecular reactions generated by mixing a mass of biomolecules with enzymes. This situation limits the experimenter’s control of the space and time of reactions. Several nanotechnology-based techniques have been developed for these reactions, including some using enzyme-functionalized microbeads^1–5^ and atomic force microscopy (AFM) tips^6–8^. These techniques allow processing to be pinpointed to a single targeted sample under a microscope at a specific time by bringing individual molecules into contact. Numerous techniques for single-molecule analysis of nucleic acids and proteins and single-cell analysis have been reported^9,10^.

However, there are some limitations to these approaches, Microbeads have poor contact with target molecules because of their spherical shape^11^. Although an AFM tip allows for precise positioning, its integration with the imaging of a target sample remains technically challenging. Physical probes such as AFM tips can only access open-to-air samples, making it impossible to use microfluidic devices for sample positioning, orientation, and shape control. To address these issues, we previously developed optically driven microstructures for efficient DNA manipulation. These microstructures allow efficient contact between DNA molecules and a structure^11^. The technique is limited to the physical manipulation of molecules including picking up, winding, and unwinding of DNA molecules^12,13^.

We expanded upon the existing functionality of an optically driven microstructure to produce a molecular processing microtool in which the geometry and layout of the surface enzymes are designed to act at a specific position on a target molecule. In this paper, we describe the development of technologies for targeted on-site cutting of single chromosomal DNA molecules as a specific application. Chromosomal DNA molecules are readily fragmented by shear forces, resulting in the loss of positional information^14^. Optical mapping techniques such as fluorescent in situ hybridization^15^ visualize specific sequences under an optical microscope, providing position references. On-site cutting of an optically mapped DNA molecule may allow the collection of DNA fragments with spatial information along the DNA, which could accelerate the development of single-molecule DNA sequencing^16^.

**Figure 1:**
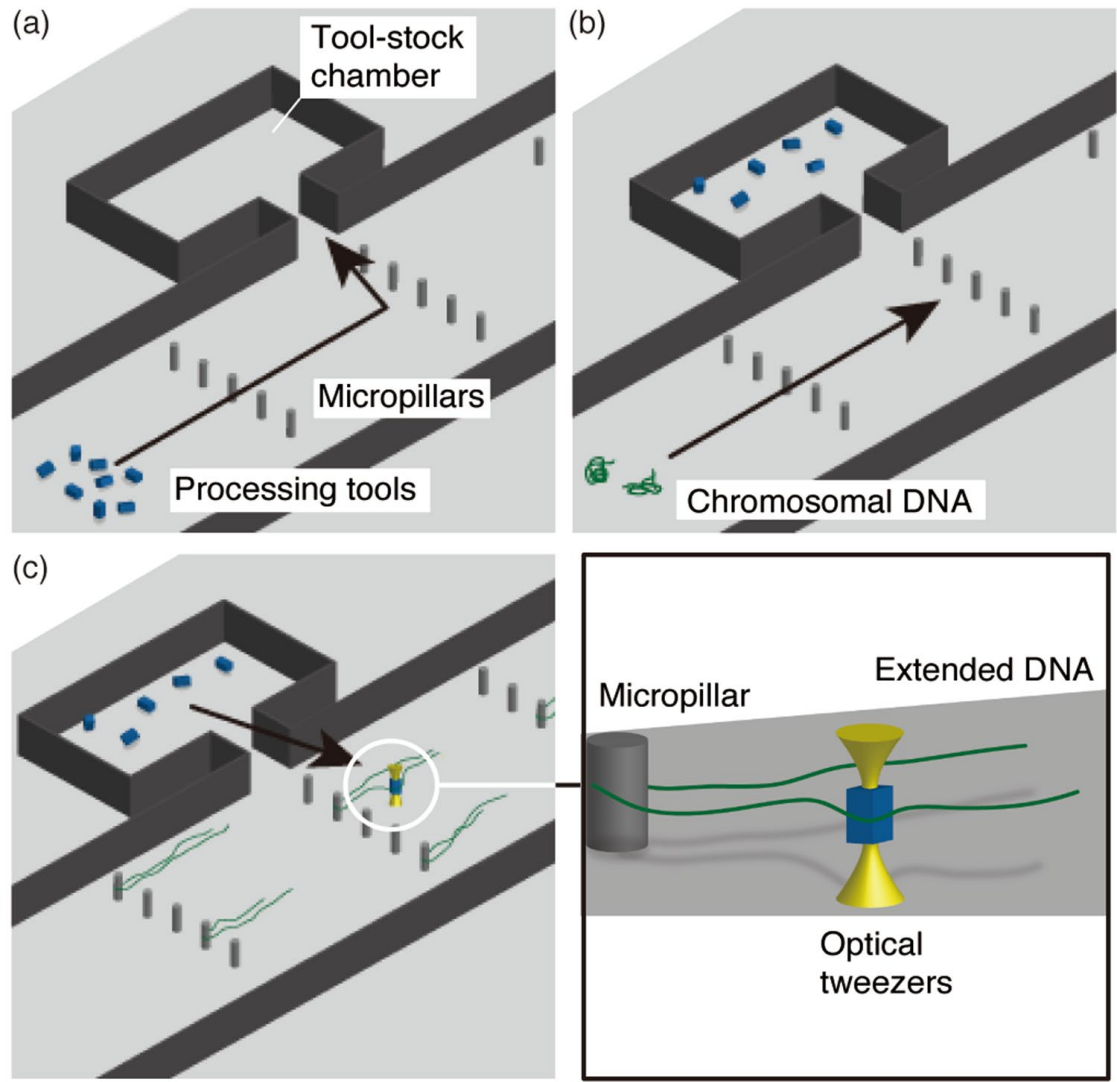
On-site processing of DNA molecules. (**a**) Filling a tool-stock chamber with microtool solution. (**b**) Introducing chromosomal DNA molecules. Micropillars trap the DNA molecules and the flow extends them. (**c**) Loading a microtool with optical tweezers showing contact between the tool and target site of an extended DNA molecule to produce an enzymatic reaction.

In our system, on-site processing is performed using microtools driven by optical tweezers in a microfluidic workbench (Fig. 1). An optically driven microtool has enzymes on its surface, enabling the induction of chemical reactions at the point of contact between the tool and target molecule. The technical hurdles for feasible space-resolved processing of single DNA molecules are the storage of microtools away from DNA molecules to minimize the occurrence of nonspecific reactions between these components and the positioning and extension of DNA molecules that exhibit random-coil conformation in aqueous solution^17^. To overcome these issues, we developed a microfluidic workbench for DNA processing that includes tool-stock chambers connected to a main flow channel in which micropillars are placed. The microfluidic device is fabricated from polydimethyl siloxane (PDMS), a material that can contain gas and has high gas permeability^18^. The gas in the PDMS device is reduced in a vacuum chamber, and then a solution containing dispersed microtools is introduced into the flow channel. Air bubbles trapped in the tool-stock chambers are gradually absorbed onto the PDMS, bringing the microtools into the chambers (Fig. 1a). The chamber structures prevent flow in the main channel from irrupting there. This zero-flow chamber has previously been used in cell trapping devices^5,19^. We used the chamber for microtool storage to form a “toolbox” for processing.

Single chromosomal DNA molecules are introduced into the channel after the microtools are washed with buffer solution. They are trapped stochastically at micropillars and extended by the flow as previously described^14,20,21^ (Fig. 1b,c). This system permits high-resolution imaging and processing under a fluorescence microscope. Optical tweezers trap one of the microtools, load it into the main flow channel, and bring it into contact with the target position of a DNA molecule to induce a chemical reaction at that point (Fig. 1c). The microfluidic workbench enables the separation of microtools from target molecules and loading of a chosen microtool into a chamber. If multiple microtools must be applied to a device, the zero flow in the chamber allows easy identification of a target microtool based on its shape or fluorescence. Thus, the system can be applied in a series of processing steps using multiple enzymes for a single sample. In this paper, we describe on-site cutting of yeast chromosomal DNA molecules using microtools on which endonucleases were immobilized.

## Results

### Microtool

**Figure 2:**
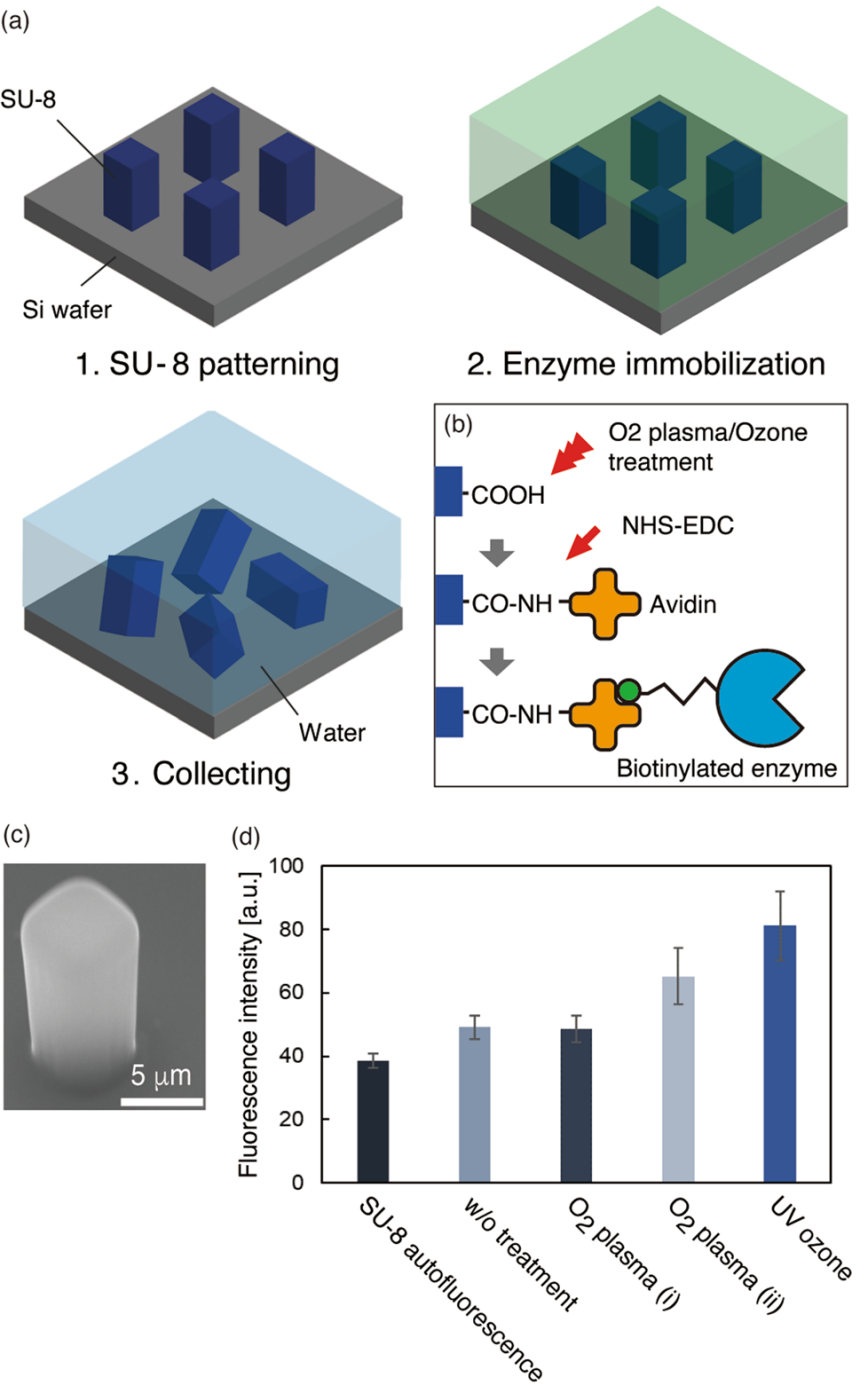
Microtools. (**a**) Process of patterning, enzyme immobilization, and collection in aqueous solution. (**b**) Surface modification for enzyme immobilization on the SU-8 surface. (**c**) An SU-8 microtool on an Si wafer. (**d**) Fluorescence intensity of microtools on which fluorescent antibodies (FITC-IgG) were immobilized using different surface treatments. Error bars: S.D. N = 10.

The fabrication process was based on photolithography and surface modification (Fig. 2a,b), which is highly scalable. Microtools were collected in aqueous solution at a concentration of 1.2 × 10^4^ pieces/µL. Each microtool was fabricated as a rectangular cuboid of 5.8 ± 0.4 µm (*x* and *y*) × 10.5 ± 0.2 µm (*z*) (mean ± S.D., Fig. 2c).

We modified tool surface using O_2_ plasma or ozone to introduce carboxyl groups^22,23^ (Fig. 2b). Avidin molecules were immobilized on the surface through their amine groups by cross coupling agents: *N*-hydroxysuccinimide and 1-ethyl-3-(3-dimethylaminopropyl) carbodiimide hydrochloride (NHS-EDC). Then, biotinylated enzymes were immobilized onto the surface through avidin–biotin bindings. Avidin–biotin system is widely used to immobilize enzymes onto a solid surface^24^, because enzymes are easy to be biotinylated, and it has less impact on the enzymatic activity^25^. Prior to the immobilization of enzymes to microtools, we conducted an experiment to evaluate chemical modifications to an SU-8 surface by immobilizing fluorescent proteins (FITC-IgG) onto SU-8 microstructures treated with O_2_ plasma (RF and LF) and ozone rather than avidin solution, and compared the results under a fluorescence microscope (Fig. 2d). Untreated SU-8 showed higher intensity fluorescence compared to the SU-8 autofluorescence, indicating physical adsorption of FITC-IgG. O_2_ plasma treatment (i) (LF, power: 100 W) showed an amount of protein similar to the physical adsorption. However, O_2_ plasma treatment (ii) (RF, power: 200 W) and UV-ozone treatment resulted in greater immobilization than physical adsorption, with the UV-ozone treatment showing the highest value.

### Flow simulation

**Figure 3:**
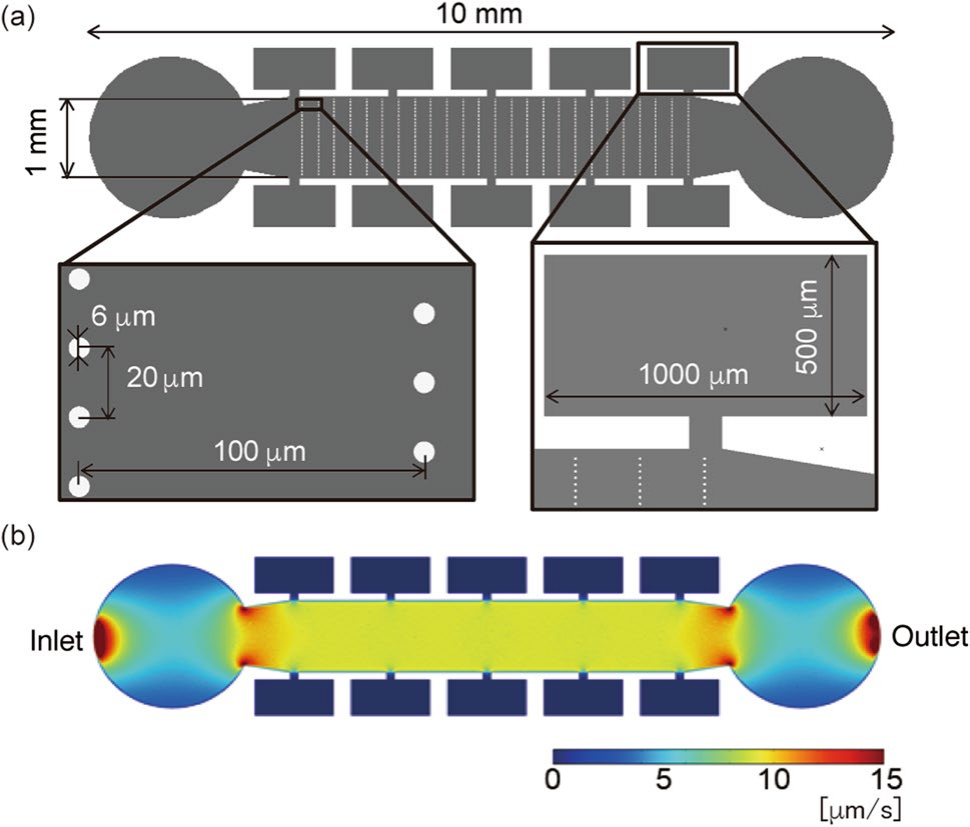
Microchannel for on-site processing. (**a**) Dimensions of the channel. (**b**) Simulation of flow velocity.

The microfluidic device has one inlet and one outlet, with 10 tool-stock chambers between them (Fig. 3a). Micropillars 6 µm in diameter were placed in the main flow channel. We checked the flow velocity by numerical simulation using the COMSOL Multiphysics 5.2a software (details are in Supplementary information). The microchannel model had a channel height of 25 μm, at which the Reynolds number was estimated to be 2.2 at a flow rate of 1 µL/min in the inlet.

The simulation results showed that the flow velocity was approximately 10 µm/s in the main flow channel and zero in the tool-stock chambers (Fig. 3b). The zero-flow region enables the storage of microtools during the processing of DNA molecules.

### Tool loading

**Figure 4:**
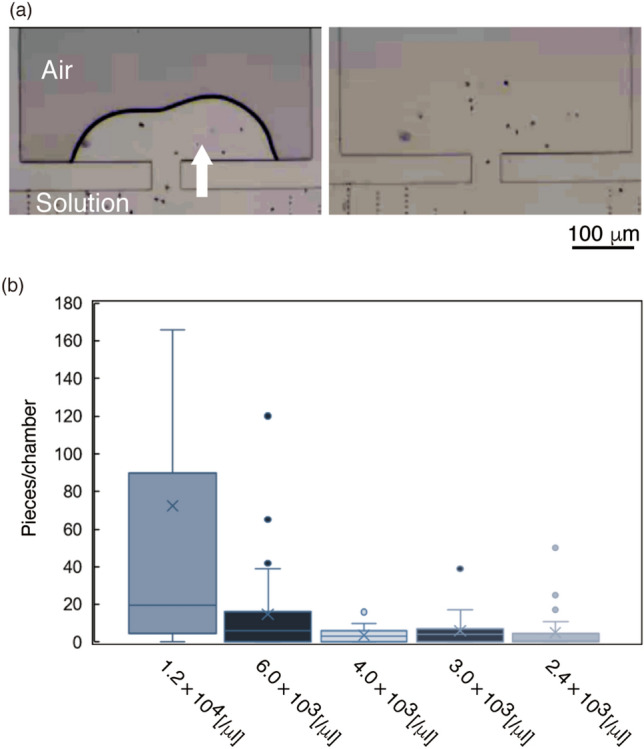
Introducing microtools into tool-stock chambers. (**a**) Process of filling with a solution containing microtools by absorption of air in PDMS. (**b**) Relationship between the number of microtools and concentration of the applied solution. × : mean, line in a box: median, box: interquartile range, whisker: 1.5 × interquartile range. N = 9.

Solutions containing microtools were applied to the PDMS microfluidic devices. The air trapped in the tool-stock chambers was absorbed into the PDMS, as previously reported^5,19^, loading microtools into the chambers (Fig. 4a). After washing out the microtools remaining in the main flow channel with PBS, we counted the number of microtools in the chambers (Fig. 4b). This count showed wide variability, although the number depended on the concentration of the microtools applied to the device. A concentration of 1.2 × 10^4^ pieces/µL solution produced the largest values and brought more than ten microtools per chamber, which is sufficient for optical manipulation, whereas the lower concentration solutions showed considerably small numbers. Thus, we used a concentration of 1.2 × 10^4^ pieces/µL solution in further experiments.

### On-site processing

**Figure 5:**
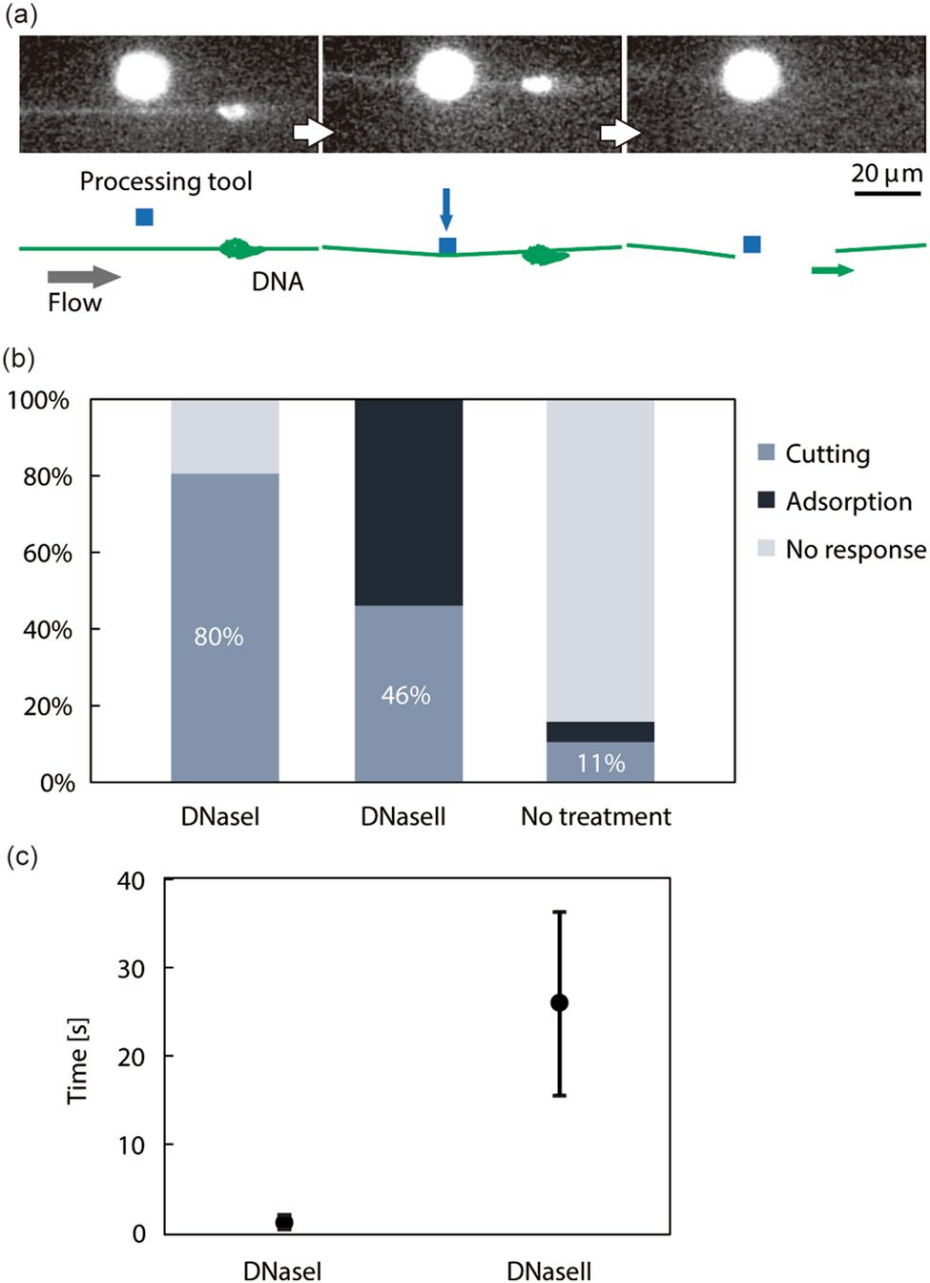
On-site cutting of single chromosomal DNA molecule with microtools on which DNA endonuclease (DNaseI and DNaseII) are immobilized. (**a**) Time series of DNA cutting. (**b**) Ratio of the reaction on a microtool with DNA. Cutting: Successful cutting of a DNA molecule at the contact position. Adsorption: Irreversible adsorption between a DNA molecule and microtool. No response: No cutting and no adsorption. N = 31 for DNaseI, N = 13 for DNaseII, N = 19 for No treatment. (**c**) Time required for DNA cutting using microtools. Error bar: S.D. N = 25 for DNaseI, N = 6 for DNaseII.

A microtool with endonucleases on its surface was moved to be in contact with a chromosomal DNA molecule extended by the flow and the DNA was cut at the contact point (Fig. 5a, Supplementary Movie S1). The DNA fragment flowed downstream, revealing the presence of a double-stranded DNA break.

We immobilized DNaseI and DNaseII endonucleases onto microtools, and compared the efficiency of processing (Fig. 5b). They have the common ability to digest DNA, though, they differ in biochemical and biological properties^26^. DNaseI microtools cut with a probability of 80%. Repeated application of the tool to the DNA molecules resulted in 100% of the molecules being cut. This observation suggests that stochastic DNaseI reactions on microtools are a reliable method of cutting. DNaseII microtools cut with a probability of 46%. The other 54% of molecules were adsorbed to the microtool at the contact point, precluding further manipulation. Microtools without enzyme modification cut 11% of the molecules.

We also evaluated the time required for DNA cutting using DNaseI and DNaseII microtools after contact with a DNA molecule (Fig. 5c). The DNaseI tools required 1.2 ± 0.8 s (mean ± S.D.) to cut a DNA molecule, whereas DNaseII tools required 25.9 ± 10.3 s.

## Discussion

Microtools were successfully fabricated with bulk processing by photolithography. The configuration for optical manipulation enabled trapping of a microtool with the longest axis, the z-axis, aligned along the direction of laser propagation. Hence, the sides of a tool that were functionalized with enzymes contacted the target molecules. The size of a tool was sufficiently smaller than the extended DNA molecules, and comparable to single cells, although it was significantly larger than single biomolecules of nanometer size, such as proteins. The size of the processing area depends on the size of the contact point of a microtool, which can be miniaturized by nano-fabrication.

Immobilization of biomolecules onto SU-8 surface have been reported, including those based on physical adsorption^27,28^ and crosslinking^29,30^. The introduction of carboxyl groups to SU-8 is better suited for on-site processing of DNA molecules than the use of amine groups. Because DNA molecules are negatively charged in a typical buffer solution, they are easily adsorbed to amine-modified surfaces, which are positively charged, through electrostatic interactions^13^. We experimentally verified the adsorption of microtools on which enzymes were immobilized through amine modification to DNA molecules without the desired enzymatic reaction. Thus, we introduced carboxy groups via dry processing (O_2_ plasma^22^ and UV-ozone^23^ treatment) which can be readily integrated into the SU-8 photolihography process. This led to the introduction of carboxyl groups using ozone treatment for on-site processing experiments.

From the results of the microtool loading, we concluded that the number of microtools is sufficient to permit manipulation by optical tweezers. In this study, we used one type of microtool that cut DNA molecules in a single experiment. When multiple tools were required for successive processing of biomolecules, a mixture of multiple microtools with different enzymes was introduced into the tool-stock chambers. A desired microtool was identified under an optical microscope based on its shape, size, and fluorescence. The flexibility in the design of microtools enables the use of different enzymes for sequential processing.

The results showed successful DNA cutting with DNaseI and DNaseII tools. Cutting by the microtools without enzyme immobilization may be caused by photodamage from the excitation light and the tension induced by elongational flow on the DNA molecule, because the exposure time in the control experiments was longer than that in the experiments with enzyme tools. The short time required for DNA cutting using a DNaseI tool can contribute the inhibition of undesired cutting. Endonucleases immobilized on a solid surface have lower enzymatic activity than free molecules^31^, although the results suggested that the immobilized molecule still functioned as an on-site DNA cutter. The results also showed that the difference in processing efficiency highly depends on the type of enzyme. DNaseI is suitable for on-site processing with 100% cutting probability through several contacts. The time required for DNA cutting also supports the superiority of the DNaseI tool. The difference of the performance between DNaseI and DNaseII tools might arise from the specificity of the enzymatic activity. Nonspecific DNA digest is desirable activity for enzymes immobilized on a DNA cutting tool. Extensive biochemical analyses have demonstrated that these two endonucleases differ in specificity: DNaseI binds across the minor grooves that exist over entire DNA length, whereas DNaseII requires a stacked single strand of limited exposure^32,33^. This indicates that the physical interaction of DNaseII with DNA imparts some digest preferences and limitations^34^. Thus, fewer bonds in DNA are susceptible to the action of DNaseII than to that of DNaseI^35^. The difference in specificity of DNA digest may reflect the efficiency and time required for on-site DNA cutting.

Although we demonstrated on-site DNA cutting, we did not obtain positional information along a DNA molecule. Cutting of a desired DNA position requires an additional technique to locate a long DNA fiber. In combination with optical mapping techniques^15^, on-site cutting may allow the collection of DNA fragments with spatial information along the DNA, which will lead to the development of single molecule genomic DNA sequencing.

The flexibility of microtool design and its surface activation will allow the processing of various samples, including biomolecules and single cells. These results suggest that the selection of enzymes, as well as the method of immobilization, is a key factor in optimization of the tools for on-site processing. On-site processing also requires control of the position, orientation, and shape of a target sample under an optical microscope, underscoring the necessity for concurrent development of the microfluidic workbench and microtools. Integrating DNA trapping^20,36,37^ and single-cell trapping structures^38–43^ with our proposed methodology may extend the range of on-site and pin-point enzymatic processing.

We developed laser-manipulated microtools for processing single biomolecules precisely under an optical microscope using enzymatic reactions. The microtools have high contact efficiency with a target molecule and allow the use of a microfluidic device for controlling the position, orientation and shape of the target sample. The microtools were fabricated by standard photolithography, their surfaces were carboxylated by ozone treatment, and enzymes were immobilized on the surfaces. We developed a microfluidic workbench for processing single chromosomal DNA molecules with the functions of tool loading and positioning and DNA extension. We demonstrated pinpoint chromosomal DNA cutting by DNaseI and DNaseII immobilized microtools, which showed differences in cutting efficiency. The pinpoint processing may provide an approach for analyzing chromosomal DNA at the single-molecule level. The flexibility of the tool and workbench design and surface functionalization will allow the application of a range of pinpoint enzyme reactions to targeted portions of single biosamples. This work provides a basis for the further development of bio-analysis techniques based on single-molecule and single-cell manipulation.

## Methods

### Microtool fabrication

We fabricated microtools using an SU-8 photoresist (MicroChem Corp., Westborough, MA, USA) (Fig. 2a). A microtool is a rectangular 5 × 5 × 10 µm cuboid. One million microstructures were fabricated on a piece of a 4-inch Si wafer divided into four. A wafer was coated with SU-8 3010 and patterned using a UV mask aligner (MA6/BA6, SÜSS MicroTec SE, Garching near Munich, Germany). After washing with isopropyl alcohol, the substrate was baked at 180 °C for 2 h.

We modified the surface using O_2_ plasma or ozone to introduce carboxyl groups^22,23^ (Fig. 2b). During O_2_ plasma treatment, a reactive ion etching apparatus (RIE-10NR, SAMCO, Inc., Kyoto, Japan) and plasma cleaner (CUTE-MP, Femto Science, Inc., Somerset, NJ, USA) were used at 20 sccm with a 20 Pa of O_2_, 200 W of output power for 30 s, and at 20 sccm with 20 Pa of O_2_, 100 W of output power for 30 s. During ozone treatment, a UV ozone cleaner (UV253V8, Filgen Inc.) was used with 20 Pa of O_2_ for 60 min. A solution of *N*-hydroxysuccinimide (Sigma-Aldrich, St. Louis, MO, USA), 1-ethyl-3-(3-dimethylaminopropyl) carbodiimide hydrochloride (Sigma-Aldrich) was then dispensed onto the wafer and incubated for 60 min. The solution was then replaced with 1 mg/mL avidin (NeutrAvidin Protein, Thermo Fisher Scientific, Waltham, MA, USA) to crosslink the amine groups of the avidins to carboxyl groups on the SU-8 surface, and the wafer was incubated for 60 min and then washed with PBS. To block unreacted residues, we added ethanolamine (Tokyo Chemical Industry Co., Ltd., Tokyo, Japan) and incubated for 30 min, and then washed with PBS. To evaluate the chemical modification, 10 g/mL FITC labeled anti-human IgG antibody (Abcam, Cambridge, UK)/HEPES was added rather than avidin solution.

We immobilized DNaseI^32^ and DNaseII^34^ (Sigma-Aldrich) endonucleases onto the microtools by avidin–biotin interactions. The enzymes were biotinylated using EZ-Link sulfo-*N*-hydroxysuccinimide-biotin (Thermo Fischer Scientific), which was applied to the avidin-modified microtools, incubated for 24 h, and washed with PBS. To release the microtools from the Si wafer, we dispensed 0.5% Tween 20, scratched the surface with a pipette tip, and transferred the solution dispersing microtools into a plastic tube.

### Microfluidic device fabrication

The microfluidic device was fabricated using a standard micromolding process for polydimethylsiloxane (PDMS, Sylgard 184, Dow Chemical Company, Midland, MI, USA). SU-8 3025 resist was coated on a Si wafer to a height of 25 µm and patterned using UV lithography. A PDMS replica was obtained from the mold at a weight ratio of 10 (base PDMS polymer):1 (curing agent). The PDMS replica was punched for the tube connection, and bonded onto a glass substrate of a thickness of 0.12–0.17 mm using O_2_ plasma treatment.

### Experimental setup and procedures

The microfluidic device was placed in a vacuum chamber to remove the air inside the PDMS, and then placed on an inverted optical microscope (IX71, Olympus, Tokyo, Japan) equipped with an xyz motorized stage and transparent plate heater (Tokai Hit Co., Ltd., Shizuoka-ken, Japan). PTFE tubes were connected to the inlet and a syringe pump (Legato111, KD Scientific, Holliston, MA, USA). The temperature of the device was measured using a thermocouple and maintained at 37 °C by the plate heater and an objective lens heater. Fluorescence images were captured using a 100 × oil-immersion objective lens (N.A. 1.40, Olympus) and an EM-CCD camera (C9100-23B, Hamamatsu Photonics, Hamamatsu, Japan).

A solution containing microtools was introduced from the inlet to fill the tool-stock chambers by the adsorption of air onto the PDMS. The solution was then replaced with PBS to flush out the microtools in the main flow channel. Chromosomal DNA molecules (S. pombe, CHEF DNA size marker, Bio-Rad Laboratories, Hercules, CA, USA) stained with 1 µM fluorescence intercalator YO-PRO-1 (Thermo Fischer Scientific) were introduced into the channel at a flow rate of 300 nL/min to be trapped stochastically at the micropillars. To cut a DNA molecule, we replaced the solution in the channel with buffer solutions: 40 mM Tris-HCl pH 7.5, 8 mM MgCl_2_ for DNaseI; and 83 mM sodium acetate pH 4.5, 20 mM NaCl for DNaseII.

To manipulate a microtool using optical tweezers, we introduced an Yb fiber laser (CW 1064 nm, PYL-5-1064.LP, IPG PHOTONICS, Oxford, MA, USA) through the objective lens, and loaded it from a tool-stock chamber to the main flow channel with the motorized microscope stage. The setup for optical tweezers is detailed in our previous papers^11,12^. The tool was in contact with an extended DNA molecule at a flow rate of 300 nL/min. We observed the reactions by florescence imaging of the DNA molecules.

## Supplementary Information

Supplementary Video.

Supplementary Information.

## Data Availability

The datasets generated during and/or analyzed during the current study are available from the corresponding author on reasonable request.
