# Supplementary material for: On-site processing of single chromosomal DNA molecules using optically driven microtools on a microfluidic workbench
Source: Sci Rep. 2021 Apr 12;11:7961. doi: 10.1038/s41598-021-87238-3 (PMC8042024; doi:10.1038/s41598-021-87238-3)
Supplement: Supplementary file 2 — Supplementary Information. [file 41598_2021_87238_MOESM2_ESM.pdf]

On-site processing of single chromosomal DNA molecules using optically driven microtools on a microfluidic workbench

Akihito Masuda[a], Hidekuni Takao[a,b], Fusao Shimokawa[a,b] and Kyohei Terao[a,b]*

[a] Department of Intelligent Mechanical Systems Engineering, Kagawa University, Takamatsu 761-0396, Japan.

[b] Nano-Micro Structure Device Integrated Research Center, Kagawa University, Takamatsu 761-0396, Japan.

*Corresponding author:
Kyohei Terao
Department of Intelligent Mechanical Systems Engineering
Kagawa University, Takamatsu 761-0396, Japan
Tel: +81 (87) 864-2346
E-mail: terao. kyohei@kagawa-u.ac.jp

Supplementary information

- **Flow Simulation**

The operation in the microfluidic workbench is in laminar-flow regime, where Reynolds number is estimated to be 2.2. The governing equations are Navier-Stokes equation and mass continuity equation for imcompressive steady flow described as

$$\boldsymbol{u} \cdot \nabla \boldsymbol{u} = -\frac{1}{\rho} \nabla p + \nu \nabla^2 \boldsymbol{u}, \quad \text{(S1)}$$

$$\nabla \cdot \boldsymbol{u} = 0, \quad \text{(S2)}$$

where ρ, ν, $\boldsymbol{u}$ and p are density, kinematic viscosity, velocity and pressure respectively. We modeled the microfluidic workbench having a uniform height of 25 μm (Figure S1). The inlet and outlet are set at the left and right ends of the figure respectively. Simulation was performed using COMSOL Multiphysics 5.2a, where the mean velocity at inlet was 1.0 μm/min and the channel wall has no-slip condition except at the inlet and outlet. Working fluid was water having the density of 999.84 kg/m^3 and kinetic viscosity of 1.74 $\times 10^{-6}$ m^2/s.

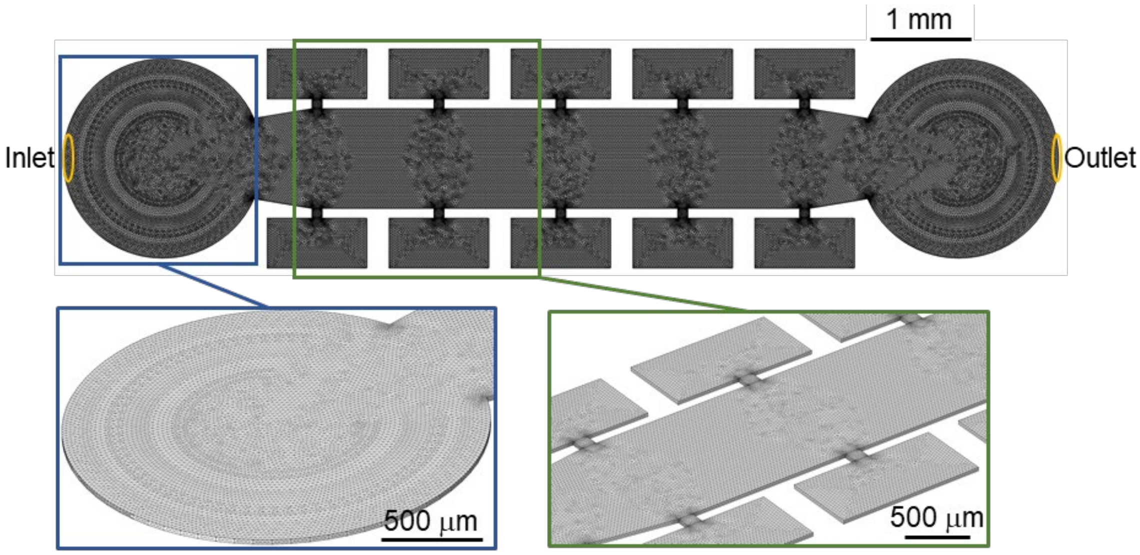

Figure S1. Simulation model of a microfluidic workbench.

- MovieS1.avi

Movie S1. On-site cutting of single chromosomal DNA molecule with microtools on which DNA endonuclease (DNaseI) are immobilized. Real-time movie.
